# Reactivation of HSV-1 following explant of tree shrew brain

**DOI:** 10.1007/s13365-015-0393-4

**Published:** 2015-10-26

**Authors:** Lihong Li, Zhuoran Li, Xin Li, Erlin Wang, Fengchao Lang, Yujie Xia, Nigel W. Fraser, Feng Gao, Jumin Zhou

**Affiliations:** Key Laboratory of Zoonosis, Ministry of Education, College of Veterinary Medicine, Jilin University, Changchun, 130062 China; Key Laboratory of Animal Models and Human Disease Mechanisms, Kunming Institute of Zoology, Chinese Academy of Sciences, Kunming, Yunnan 650223 China; Kunming Primate Research Center, Kunming Institute of Zoology, Chinese Academy of Sciences, Kunming, Yunnan 650223 China; Center for Drug Safety Evaluation, Kunming Institute of Zoology, Chinese Academy of Sciences, Kunming, Yunnan 650223 China; Department of Microbiology, Perelman School of Medicine, University of Pennsylvania, Philadelphia, PA 19104 USA

**Keywords:** Tree shrew, HSV-1, Latency, Reactivation, CNS, Explant cocultivation

## Abstract

Herpes Simplex Virus type I (HSV-1) latently infects peripheral nervous system (PNS) sensory neurons, and its reactivation leads to recurring cold sores. The reactivated HSV-1 can travel retrograde from the PNS into the central nervous system (CNS) and is known to be causative of Herpes Simplex viral encephalitis. HSV-1 infection in the PNS is well documented, but little is known on the fate of HSV-1 once it enters the CNS. In the murine model, HSV-1 genome persists in the CNS once infected through an ocular route. To gain more details of HSV-1 infection in the CNS, we characterized HSV-1 infection of the tree shrew (*Tupaia belangeri chinensis*) brain following ocular inoculation. Here, we report that HSV-1 enters the tree shrew brain following ocular inoculation and HSV-1 transcripts, ICP0, ICP4, and LAT can be detected at 5 days post-infection (p.i.), peaking at 10 days p.i. After 2 weeks, ICP4 and ICP0 transcripts are reduced to a basal level, but the LAT intron region continues to be expressed. Live virus could be recovered from the olfactory bulb and brain stem tissue. Viral proteins could be detected using anti-HSV-1 antibodies and anti-ICP4 antibody, during the acute stage but not beyond. In situ hybridization could detect LAT during acute infection in most brain regions and in olfactory bulb and brain stem tissue well beyond the acute stage. Using a homogenate from these tissues’ post-acute infection, we did not recover live HSV-1 virus, supporting a latent infection, but using a modified explant cocultivation technique, we were able to recover reactivated virus from these tissues, suggesting that the HSV-1 virus latently infects the tree shrew CNS. Compared to mouse, the CNS acute infection of the tree shrew is delayed and the olfactory bulb contains most latent virus. During the acute stage, a portion of the infected tree shrews exhibit symptoms similar to human viral encephalitis. These findings, together with the fact that tree shrews are closely related to primates, provided a valuable alternative model to study HSV-1 infection and pathogenesis in the CNS.

## Introduction

Following eye infection, Herpes simplex virus type 1(HSV-1) is transferred by fast axonal transport to the nuclei of trigeminal ganglia sensory neurons, in which it forms a latent infection. Reactivation of this latent infection leads to productive viral infection at the peripheral site of inoculation, which can cause skin lesions and herpes simplex keratitis (HSK). The reactivated virus can also spread to the central nervous system (CNS), where it may lead to Herpes Simplex Virus encephalitis (HSE), the most common cause of sporadic and often fatal encephalitis (Kastrukoff et al. 1981; Lundberg et al. 2008). Studies suggest that HSE pathogenesis is related to the reactivation of latent virus in the brain tissue, rather than the spread of reactivated virus from ganglia (Sekizawa and Openshaw 1984; Jones et al. 2005; Chen et al. 2006). With an incidence of 1 in 200,000 individuals per year, HSE is associated with 70 % mortality in untreated patients and 30 % mortality in treated patients, leaving most affected individuals permanent neurological damages (Kimberlin 2003; Chayavichitsilp et al. 2009). HSV-1 genomes can be detected in the human brain (Fraser et al. 1981; Lin et al. 1997), and both primary and recurrent infections may induce encephalitis (Gilden et al. 2007), with the latter accounting for 70 % of encephalitis cases (Chaudhuri and Kennedy 2002). However, the mechanism of HSE pathogenesis is not well understood. Clearly, HSV establishes latency in sensory ganglia of the peripheral nervous system (PNS) (Spivack and Fraser 1987; Spivack et al. 1995; Webre et al. 2012; Roizman and Whitley 2013), but whether HSV exists as latent or permanently silenced viral genome in the CNS has not been fully investigated (Cabrera et al. 1980; Deatly et al. 1988). Mouse and rabbit CNS HSV-1 infections have been reported (Knotts et al. 1973; Rock and Fraser 1983; Smith et al. 2000). Recent evidence demonstrating that HSV-1 could be reactivated using a modified explant co cultivation method suggests that HSV-1 can form latent infections from which it can be reactivated in the CNS (Chen et al. 2006).

Although rodent models have been used extensively to study HSV infection, they differ significantly from human infection and pathogenesis due to their distance from primates in evolution and their differences in immunity. In addition, significant differences also exist between murine and rabbit latency infection models (Nicoll et al. 2012; Webre et al. 2012), raising a need for an alternative model that is both economic and more closely mimics the human HSV infection. The tree shrew is a small squirrel-like animal indigenous to southwest Asia and is more closely related to primates than do rodents at both genomic and transcriptomic levels (Fan et al. 2014; Xu et al. 2013a; Zheng et al. 2014). Anatomically, a tree shrew brain is more similar to a primate’s (Wang et al. 2013), supporting the development of tree shrews as alternative human disease models, including the study of vision, social stress, and neurological/psychiatric diseases, to name a few (Ohl et al. 1998; Guo et al. 2013; He et al. 2014).

HSV has been known to infect and establish latent infections in tree shrews since the 1970s (Darai et al. 1978; Xu et al. 2013b). Several studies have been done using an intraperitoneal infection (Rosenwolff et al. 1989). In this study, we have further characterized HSV-1 infection of the tree shrew CNS following ocular inoculation. During the acute stage of infection, live virus could be first detected on 5 days post-infection (p.i.), peaked on 10 days p.i., and disappeared after 2 weeks p.i. Coincident with this, viral transcripts ICP0, ICP4, and LAT intron, detected by qRT-PCR, also started to appear on 5 days p.i., peaked between 8 and 10 days p.i., and returned to a basal level with the exception of LAT intron, which remain at a high level of expression for the entire course of our observation (2 months). We also detected HSV-1 antigens by immunohistochemistry (IHC) using anti-HSV-1 antibodies and ICP4 protein immunofluorescence (IF) using anti ICP4 antibody. The expression of LAT after the acute stage infection led us to hypothesize a latent infection by HSV-1. In situ hybridizations analysis using a stable LAT intron confirmed that LAT is indeed expressed in brain stem and olfactory bulb well beyond acute stage. Using explant cocultivation experiment, we were able to recover reactivated virus from brain stem and olfactory bulb. Taken together, these results demonstrated that HSV-1 latently infects the CNS of tree shrew.

## Materials and methods

### Animals

Adult tree shrews (Tupaia belangeri chinensis), 6-month-old females, were obtained from the animal breeding facility at the Kunming Institute of Zoology (KIZ). BALB/c mice were used as experimental controls and were raised in the SPF facility in KIZ.

### Ethics statement

All tree shrew and mouse experiments were performed according to the procedures and policies of “The Guide for the Care and Use of Laboratory Animals” (published by the NATIONAL ACADEMY PRESS Washington, D.C.1996). The study protocol of ocular HSV-1 infection was reviewed and approved by the Institutional Animal Care and Use Committee of Kunming Institute of Zoology, Chinese Academy of Sciences. The identification number is SYDW20121201001.

### Handling of animals

Animals were anesthetized with an intramuscular injection with ketamine (100 mg/kg), and infection achieved by dropping 10^6^ PFU of HSV-1 McKrae virus inoculum on each eye without ocular scarification. Mock-infected animals were anesthetized, and a PBS solution of equal volume was dropped on each eye. Animals were returned to cages following recovery from the anesthesia and monitored every 12 h during the first week, and daily during the second week following infection, for signs of disease.

#### Viruses

The pathogenic HSV-1 strain McKrae was used in our experiments. All virus was grown on vero cells and titered on RS1 cells.

#### Cell lines and antibodies

All cells were obtained from Conservation Genetics CAS Kunming Cell Bank and were grown in T75 flask (NEST, 708001) with Dulbecco’s Modified Eagle Medium (Gibco, 12800–017) and 5 %(*v*/*v*) fetal bovine serum (Pufei, 1101–500). These cells were used to prepare the viruses and determine virus titer. Rabbit skin cells (RS1) cultured in same conditions were used for brain explant cocultivation experiments. The ICP4 monoclonal antibody was obtained from Gerd Maul’s laboratory at the Wistar Institute (Showalter et al. 1981). HSV-1 polyclonal antibodies were from American Qualex Antibodies (RR1190P).

### HSV-1 titer in mouse and tree shrew brains during acute infection

To assay for the presence of infectious virus in mice or tree shrew’s brain during the acute stage of infection, parts of the brain were homogenized and the supernatant incubated with RS1 cells to detect infectious virus. Viral titering was done according to Chayavichitsilp et al. (2009). We dissected and homogenized the brain from infected animals at mock-infected, 3, 5, 8, 10, and 13 days post-infection, followed by coculturing the supernatant with RS1 cells to detect live HSV-1 virus.

### HSV-1 viral genomic DNA load in latently infected mouse and tree shrew brain tissue determined by Quantitative real-time PCR

Mice or tree shrews brain stems and olfactory bulbs were harvested from infected animals 30 days post-infection. The genomic DNA was extracted by Genomic DNA purification kit (DP304-03, Tiangen), and the amounts of viral (UL30, DNA polymerase) and cellular (18 s) DNA in samples were quantified by real-time PCR. The quantification standards of viral genomes were prepared by reconstituting known amounts (copies) of HSV-1 genomes with homogenates of brain stems or olfactory bulbs from uninfected mice or tree shrews. Known amounts of tissue DNA (in μg) prepared from mouse or tree shrew brain stems and olfactory bulbs were used as the quantification standards for cellular DNA. The number of copies of viral genomes was normalized to the amount of cellular DNA in the sample and expressed as viral genome copies per microgram of tissue DNA.

### Total RNA extraction from tissue

At 4 weeks post-infection (p.i.), animals were euthanized, and the brains dissected and prepared for qRT-PCR. Brain tissue frozen with liquid nitrogen was ground to a powder. Fifty milligrams of powder was placed into a 1.5-ml centrifuge tube with the addition of 1 ml Trizol. After mixing and standing for 5 min at room temperature (RT), 0.2 ml chloroform was added with vibrating for 15 s. After standing 2 min at RT, the mix was centrifuged at 4 °C, 12,000*g* × 15 min. Isopropanol (0.5 ml) was added to the supernatant followed by gently mixing, standing for 10 min at RT, and centrifuging at 4 °C and 12000 g for 10 min. The supernatant was discarded, and 1 ml 75 % ethanol was added to gently wash the precipitate. This was then centrifuged at 4 °C, 12000 g × 10 min, and the supernatant was discarded. After drying, the precipitate was dissolved in DEPC-treated H_2_O. After reading the 260/280 nm and 260 nm absorption reading, the sample was store frozen at −80 °C.

### qRT-PCR detection of HSV-1 transcripts in infected mouse and tree shrew brain

Reverse transcription from total RNA extraction to cDNA was done using PrimeScript™ RT reagent Kit with gDNA Eraser (Takara, Cat. No. RR047A). Quantitative PCR was done in a 7900HT instrument from Applied Biosystem using a FastStart Universal SYBR Green Master (Rox kit, Roche, Cat. No. 04913850001). The reference genome for HSV-1 coordinates is from HSV-1 17+, GeneBank: JN555585.

### Immunohistochemistry detection of HSV-1 proteins

Tree shrew brains were embedded in paraffin and sectioned at a thickness of 5 μm on a microtome (Leica, RM2245). Slides were treated at 60 °C for 30–60 min, then deparaffinized in xylene and a gradient concentration of ethanol. Deparaffinized slides were rinsed with water and antigen retrieved in boiling citric acid buffer (PH = 6.0) for 24 min, which was boiled by microwave. After natural cooling, slides were washed sufficiently with water, immersed in 3 % H_2_O_2_ for 10 min, and permeabilized with 0.5 % TritonX-100 at RT for 10 min. Blocked in 5 % BSA at RT for 30 min, then incubated with diluted primary antibody (Rabbit anti HSV1, Cat. RR1190P, American Qualex Antibodies) at 4 °C, incubated overnight and subsequently diluted secondary antibody added (anti-rabbit IgG HRP-labeled Antibody, Cat #7074S, Cell Signaling Technology) at room temperature for 60 min. After development with DAB (Cat. DAB-0031) for 3 min, slides were washed in water and did counterstain with hematoxylin. Slides were dehydrated in a gradient concentration of ethanol and dehydrated with xylene, for 3–5 min each. Finally, slides were mounted with neutral balsam and the signal detected with a Nikon, 80i fluorescent microscope.

### Immunofluorescence detection of ICP4 proteins

Tree shrew brains were embedded in paraffin and sectioned 5 μm thick on a microtome (Leica, RM2245). Slides were heated at 60 °C for 30–60 min, then deparaffinized in xylene and a concentration gradient of ethanol. Deparaffinized slides were rinsed with water and antigen renatured by boiling (microwave) in sodium citrate buffer at PH = 6.0 for 24 min, which was boiled by microwave. After natural cooling, slides were washed sufficiently with water (weak water flow 5 min, 3 times), and the tissue permeabilized with 0.5 % TritonX-100 at RT for 10 min. Slides were blocked in 5 % BSA at RT for 30 min, then incubated with diluted (1:1000) primary antibody (monoclonal antibody against ICP4 obtained from Dr. Gerd Maul’s laboratory) at 4 °C overnight and subsequently added diluted secondary antibody (1:1000, Alexa Fluor 594 Goat Anti-Mouse IgG (H + L) from Life Technologies) at RT for 60 min. To visualize the cell nucleus, all samples were double stained with Hoechst. Finally, slides were mounted with mounting medium and signals detected under the microscope (Nikon, 80i).

### In situ hybridization

RNA probes corresponding to the HSV-1 Latency Associated Transcript (LAT) intron region (probe position: 120,384–121,418 nt) were labeled with DigU (DIG RNA Labeling Mix, Cat.11 277 073 910, Roche). Dissected tree shrew’s brains were embedded in paraffin and sectioned 5 μm thick on a microtome (Leica, RM2245). Sections were deparaffinized in xylene and a gradient concentration of ethanol. RNA in situ hybridization was performed as followed. Briefly, slides were fixed in 4 % PFA at room temperature before protease K treatment and re-fixing in 4 % PFA. After fixation, slides were acetylated by 0.25 % acetic anhydride (sigma, 320102)/0.1 M triethanolamine (sigma, 90279). The slides were then treated in pre-hybridization solution (10 mM Tris, 600 mM NaCl, 1 mM EDTA, 0.25 % SDS, 1 × Denhardt’s, 50 % formamide, 300ug/ml Yeast tRNA) for 2 h at 60 °C and subsequently hybridized in hybridization buffer (pre-hybridization plus 339.5 μl 25 % dextran sulfate and add probe to a final concentration of 1–2 μg/ml hybridization solution) with LAT probe over night at 60 °C. After a serial wash of SSC buffers of different concentrations (1 × SSC/50 % formamide, 2 × SSC, and 0.2 × SSC buffer) at 65 °C, the Anti-Digoxigenin-AP Fab Fragments antibody (Roche, Cat. 11 093 274 910) is incubating in MABT buffer over night at 4 °C. Then, slides were developed in alkaline phosphatase buffer (100 mM Tirs PH 9.5, 50 mM MgCl_2_, 100 mM NaCl, 0.1 % Tween 20) with BCIP/NBT (Roche, Cat.11383221001, Cat.11383213001, 100 mg/ml NBT 3.375 μl, and 3.5μl 50 mg/ml BCIP in 1 ml alkaline phosphatase) and hematoxylin & eosin counterstaining. Finally, the sections were inspected using a Nikon 80i fluorescent microscope to determine presence of LAT.

### HSV-1 reactivation detection by explant cocultivation test

We used method described by Chen et al. (2006). Briefly, at 4 weeks post-infection (p.i.), animals were euthanized and brains were dissected. Brain tissues were cut into small section and incubated in serum-free DMEM containing 0.08 % trypsin, 0.013 % collagenase type I-A(Sigma), 20 mM HEPES, l-glutamine, HCO_3_, penicillin, streptomycin, and amphotericin B. After constant agitation for 40 min at 37 °C, the suspension was centrifuged at 300×*g* for 10 min at 4 °C, and the pellet was resuspended in DMEM containing 10 % fetal bovine serum, 2 mM l-glutamine, 0.075 % HCO3, 200 U/ml penicillin, 200 ug/ml streptomycin, and 0.25 ug/ml amphotericin B (Fungizone). Then, the suspension was transferred to confluent monolayers of RS1 cells (1 × 10^5^ cells/well in six-well plates). The explant cultures were incubated at 37 °C in 5 % CO_2_ with change of fresh medium every 3 days. Cultures were observed daily for the development of cytopathic effect (CPE) in RS1 cells. If no CPE was detected after 2 weeks, the brain tissue was scored negative for reactivation. The samples that showed negative CPE were further incubated for up to a month to ensure that there was indeed no CPE developing. If virus-induced CPE was noted, the cell media was removed and saved for DNA extraction and testing (PCR/sequence) for HSV-1.

To distinguish between persistent infections and latency, brain tissues were also homogenized, centrifuged at slow speed, and the supernatant incubated with RS1 cells for over 10 days to detect infectious virus. A total of 18 mouse olfactory bulbs, 18 mouse brain stems, 18 tree shrew olfactory bulbs, and 18 tree shrew brain stems were homogenized to assay for the presence of infectious virus by plaque assay on RS1 cells as previously described (Tullo et al. 1982).

## Results

### Survival rate of mice and tree shrews after ocular HSV-1 infection

**Fig. 1:**
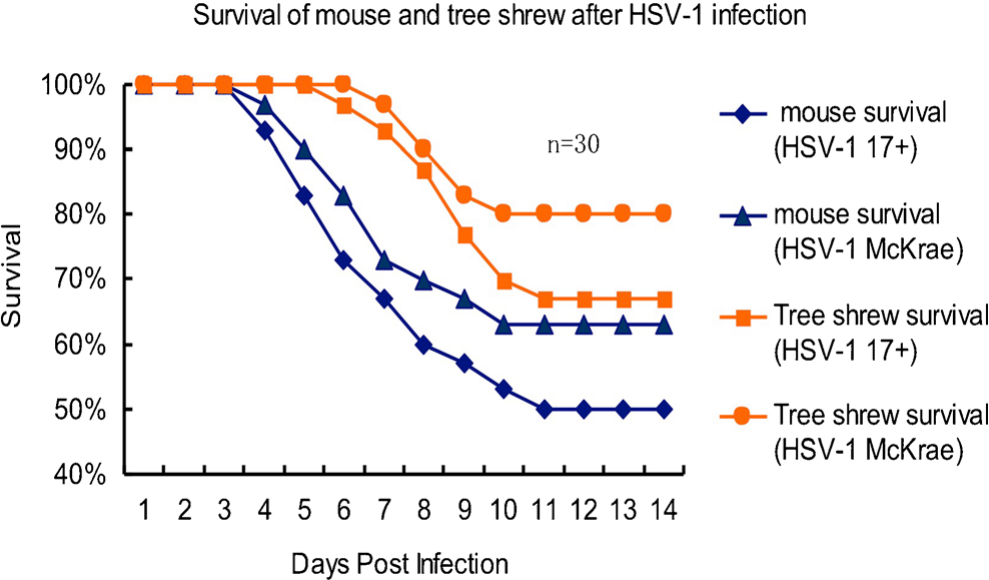
Survival rate of mice and tree shrews after HSV-1 infection. HSV-1 17+ and McKrae virus strains were inoculated into mice and tree shrews cornea, respectively. Animals were monitored daily for signs of disease and mortality. *Blue rhombus* represents HSV-1 17+ infected mice, survival rate (50 %); *blue triangle* represents HSV-1 McKrae infected mice, survival rate (63 %); *orange square* represented HSV-1 17+ infected tree shrews, survival rate (67 %); *orange circles* represented HSV-1 McKrae infected tree shrews, survival rate (80 %). *n* = 30 for each group

To determine the most humane method to infect tree shrews, we compared the lethality rate of HSV-1 17^+^ strain, applied with ocular scarification (a method typically used in rodent studies), and the more virulent McKrae strain, applied in the absence of ocular scarification, to infect both tree shrew and mouse. Tree shrews were anesthetized with ketamine, followed by ocular scarification, then 1 × 10^6^ PFU of HSV-1 17^+^ virus in PBS solution was applied to each eye. The HSV-1 McKrae virus inoculum was dropped directly onto the eyes of animals without corneal scarification. Through the same method, we infected mice, with 4 × 10^4^ PFU of HSV-1 17^+^ or McKrae virus to each eye. Eyes and other sites were monitored daily for disease signs, and the mortality was recorded (Fig. 1).

Infected tree shrews showed ruffling of fur, anorexia, weight loss, lethargy, and lack of activity during the acute stage of infection, which typically started at 5 days p.i. and lasted until the end of 2 weeks p.i.. The infected tree shrews also developed eye disease and cornea infection, which occurred from 5 days p.i. and lasted until approximately 4 weeks (the eye disease will be discussed in a separate study). About 10 % of the tree shrews showed severe nervous system disease symptoms similar to human encephalitis, such as ataxia, astasia, torticollis, looking up at the sky (or star gazing), and other abnormal behaviors. These symptoms suggest that HSV-1-infected the CNS of tree shrew during acute stage. Most of the animals displaying these symptoms perished within the first 2 weeks of the experiment (Fig. 1).

During acute infection, the clinical symptoms of tree shrews were milder than that of mice, while infected animals without ocular scarification (both tree shrew and mice) displayed milder disease symptoms than those with corneal scarification, even though the McKrea strain is known to be more virulent. Mortality of infected tree shrews occurred at around 6 and 7 days p.i., while infected mice began to die at 4 days p.i. The survival rate of HSV-1 17^+^ infected mice with ocular scarification was 50 %, while the survival rate of HSV-1 McKrae-infected mice without ocular scarification was 63 %. When tree shrews were analyzed, the survival rate of HSV-1 17^+^ infected tree shrews with ocular scarification was 67 %, but the survival rate of HSV-1 McKrae-infected tree shrews, in the absence of ocular scarification, reached up to 80 % (Fig. 1). Overall, the HSV-1 McKrae-infected animal showed better survival rate than HSV-1 17^+^ infected animals, suggesting that the scarification technique permitted a stronger infection than the use of a more virulent strain. For this reason, we chose HSV-1 McKrae strain to infect tree shrews and mice in our subsequent studies.

### HSV-1 virus was detectable in tree shrew brain during the acute stage of infection

**Fig. 2:**
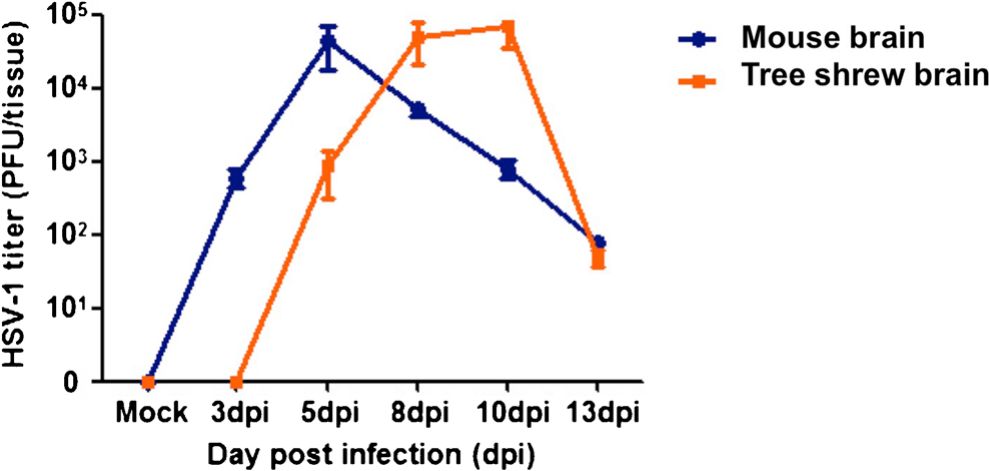
Virus titering of infected mouse and tree shrew brain. Dissected and homogenized brain supernatants from infected animals were titrated at indicated times p.i. Results are expressed as the log10 mean PFU/100 mg brain tissue for each group. Data were collected from at least two independent experiments, and the number of animals assayed at each time point (mock-infected, 3, 5, 8, 10, and 13 days p.i.) was 4. *Blue rhombus* represents viral titer in mouse brain; *orange square* represents viral titer in tree shrew brain

To confirm that a productive infection existed in HSV-1-infected tree shrew brains, we dissected and homogenized the brains from mock and infected animals at 3, 5, 8, 10, and 13 days p.i. Following a low-speed spin to pellet the cell debris, the supernatant was co-cultured with RS1 cells to detect infectious HSV-1 virus. At 20 h after incubation with supernatant from brains of infected mice and tree shrews, we could readily observe CPE due to HSV-1 lytic infection in RS1 cells. We measured the titer of the viral yield in mouse and tree shrew brain homogenate supernatants from groups of 4 animals at each time point (mock-infected, 3, 5, 8, 10, and 13 days p.i., see Fig. 2). The amount of infectious HSV-1 virus in brains of infected mice was similar to previously published data, peaking at 5 days p.i. and becoming weaker at 8 days p.i. (Yao et al. 2014). Infectious virus was detected in the infected tree shrew brain tissue at 5, 8, 10, and 13 days p.i., with the highest level of virus titer at 10 days p.i.. Unlike mouse, no CPE was seen in RS1 cells cultured with supernatant from 3 days p.i. tree shrew brain, even after extended incubation over 10 days. This result suggests that there was a delay in the acute infection of tree shrew brain compared to mouse brain following eye infection (Fig. 2).

### qRT-PCR detection of HSV-1 transcripts in acutely infected tree shrew brain

**Fig. 3:**
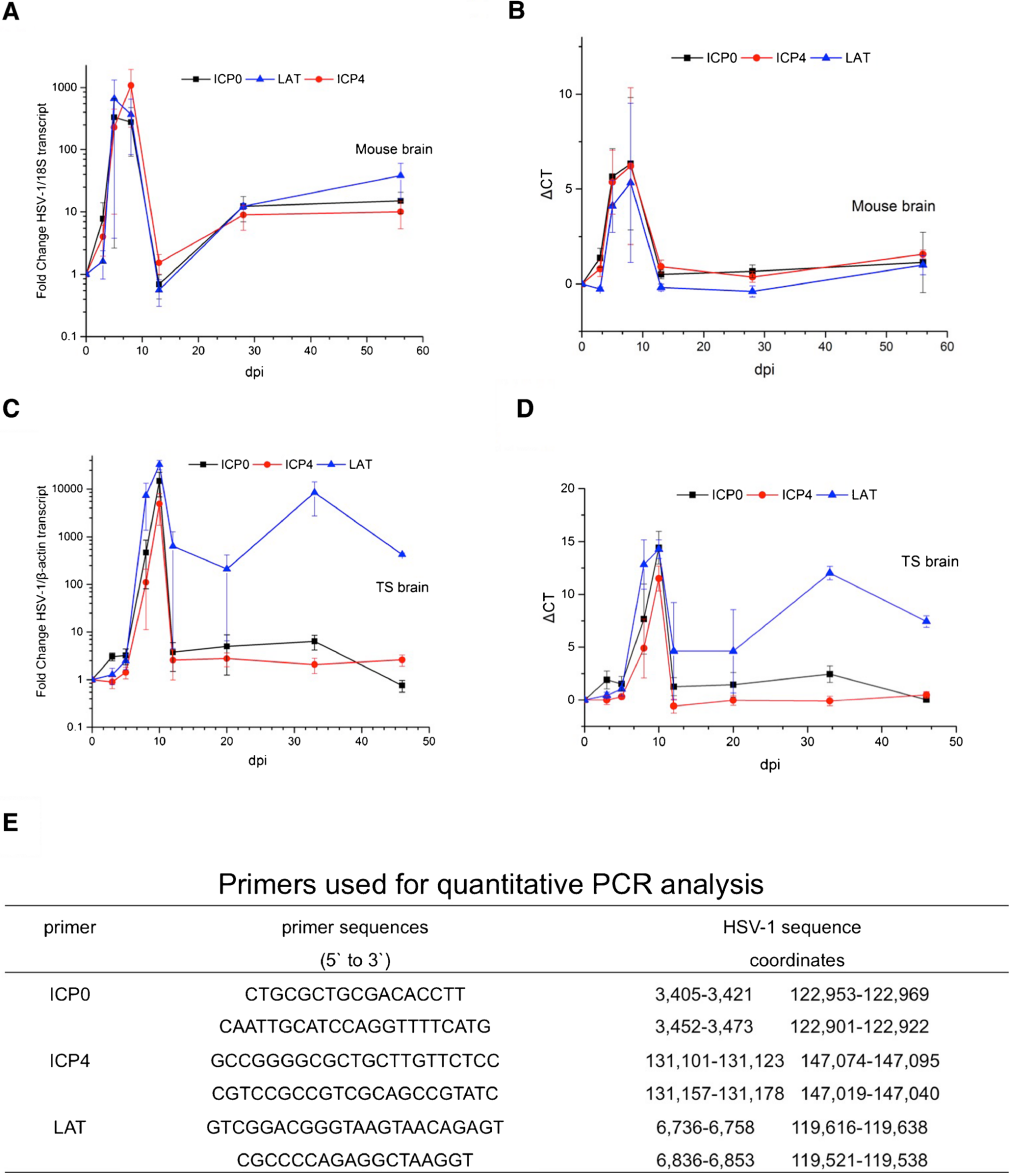
qRT-PCR detection of HSV-1 transcripts in infected animal brain. HSV-1 strain McKrae infected mice and tree shrews were anesthetized, euthanized, and dissected for brain tissue. RNA from these samples was extracted for qRT-PCR analyses. **a** Mouse brain from various days post-infection, qRT-PCR of ICP0 (*black line*), ICP4 (*red line*), and LAT (*blue line*) results were plotted as fold-change relative to 18S. **b** Similar to **a**, but the *Y*-axis was replaced with ΔCT. **c** Tree shrew brain viral transcripts were detected by qRT-PCR, and the results were represented as fold-change over β-actin. **d** Similar to **c**, except the *Y*-axis was replaced with ΔCT. **e** Primers used in the qRT-PCR detection of viral transcripts

To further investigate the HSV-1 acute infection in tree shrew brain, we monitored the expression of ICP0, ICP4, and LAT intron in tree shrew CNS tissue by qRT-PCR. For comparison, we used specific primers towards these genes to measure the respective RNA levels by qRT-PCR in HSV-1 McKrae-infected BALB/c mice. We detected high levels of transcripts from all three genes at 3 days p.i. and even higher levels at 5 days p.i., while after 2 weeks (13 days p.i.), these transcripts dropped to a low basal level (Fig. 3a, b). This pattern suggests that these mice were going through acute infection. Compared to mice, HSV-1 McKrae-infected tree shrew CNS tissue expressed ICP0, ICP4, and LAT transcripts at similar levels during acute infection, but they peaked at a later time of 10 days p.i. After 2 weeks, the expression of ICP0, ICP4 transcripts decreased to low levels; however, LAT intron continued to be expressed at higher levels (Fig. 3c, d). In mice after 20 days p.i., we did not detect significantly higher LAT expression by qRT-PCR, but we did detect LAT by in situ hybridization well beyond the acute stage.

### HSV-1 antigens were detectable using immunohistochemistry in tree shrew brain

**Fig. 4:**
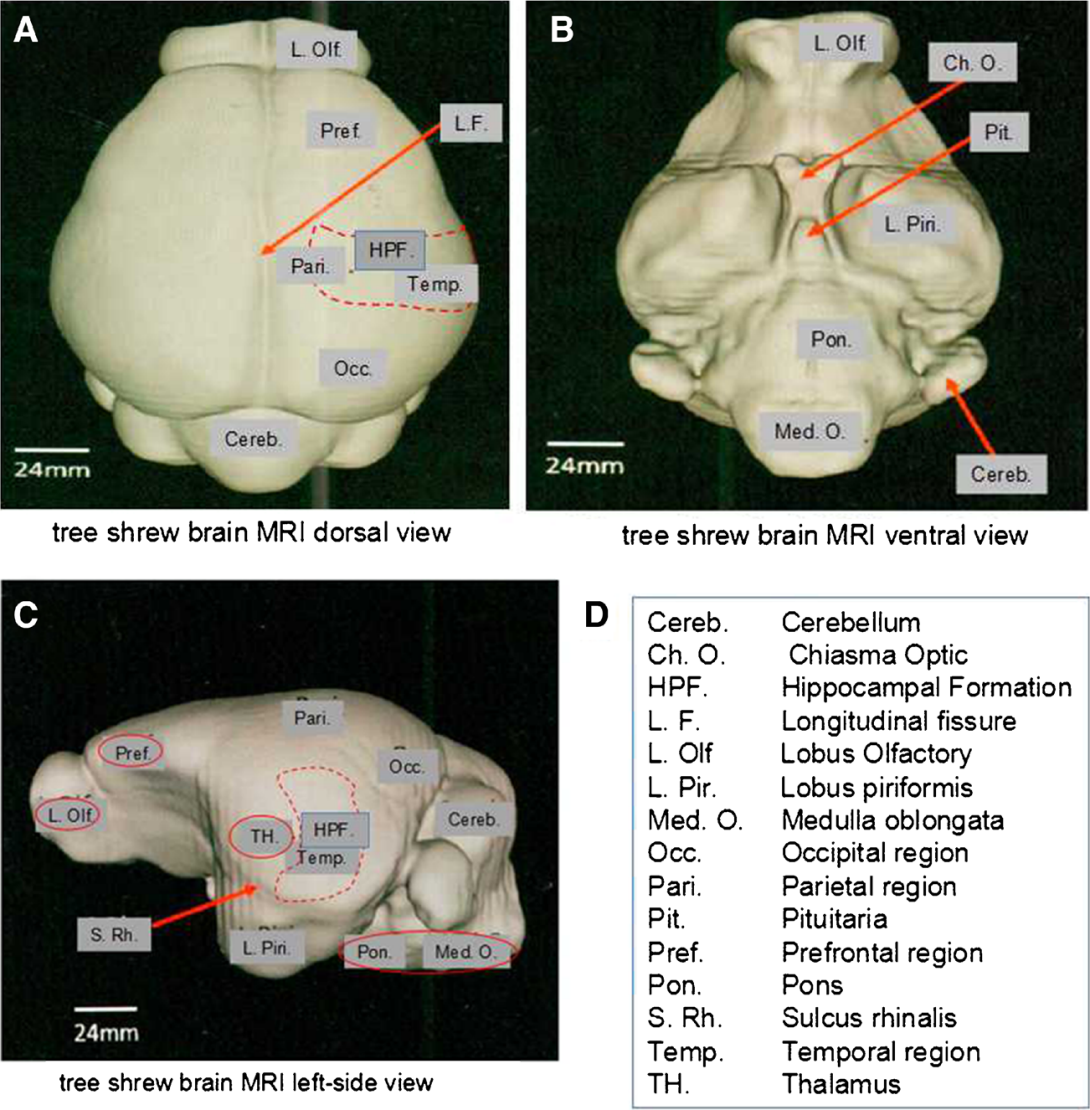
3-D view of the tree shrew brain. (adapted from Basic Biology and Disease Models of Tree Shrews (Zheng et al. 2014). Copy right release obtained from Kunming Institute of Zoology, Chinese Academy of Sciences). **a** Tree shrew brain MRI 3-D reconstruction dorsal view. **b** Tree shrew brain MRI 3-D reconstruction ventral view. **c** Tree shrew brain MRI 3-D reconstruction left-side view. **d** Different regions of the brain are marked. Dissected regions of the brain for experiments described in this study are *circled in red. Dashed circled areas* (HPF) marked areas that are beneath the surface of the brain

**Fig. 5:**
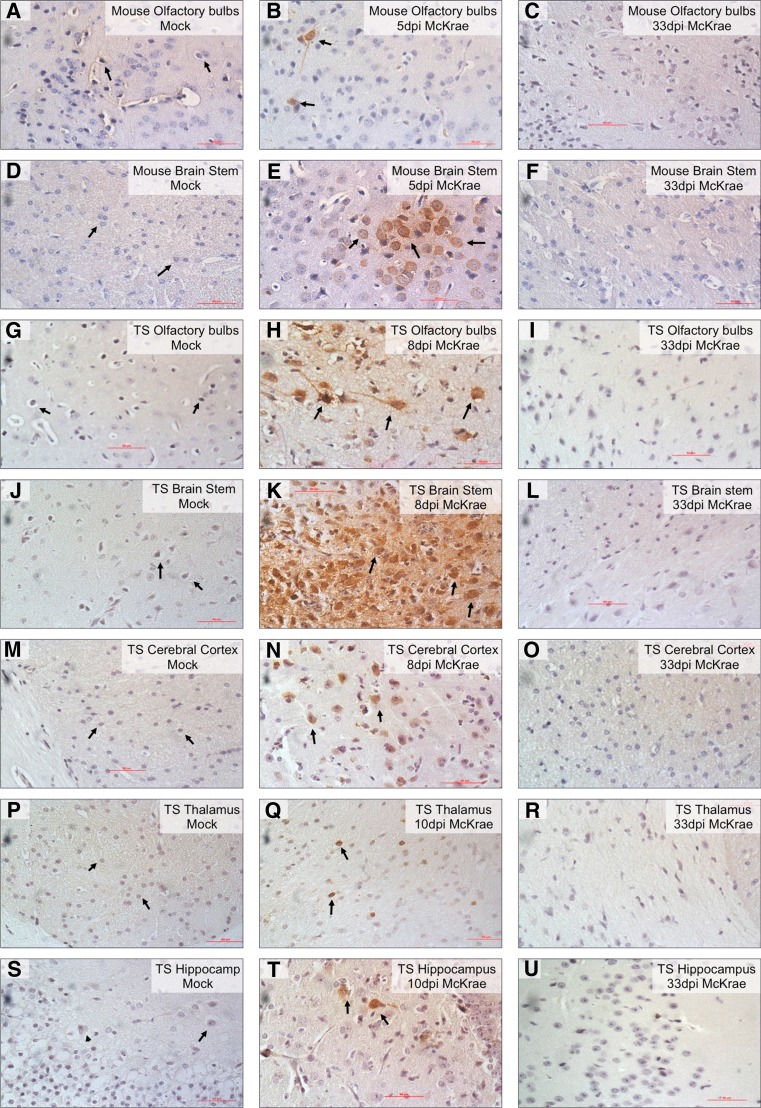
Immunohistochemical detection of HSV-1 antigens in mice and tree shrew brains. Mice and tree shrew were corneal inoculated with the McKrae strain of HSV-1 without ocular scarification. CNS tissues were dissected, paraffin embedded, sectioned, and processed for immunohistochemistry with polyclonal anti-HSV-1 antibodies. **a** and **d** Mock-infected mouse control, *arrows* point to neurons and non-neuronal cells in brain. **b** and **e** Mouse olfactory bulb and brainstem 5 days p.i. with HSV-1 McKrae, *arrows* point to infected neurons positive for HSV-1 antigens. **c** and **f** Mouse olfactory bulb and brainstem 33 days p.i. with HSV-1 McKrae, no positive signal for HSV-1 antigens. **g** Tree shrew (TS) olfactory bulb mock-infected control, *arrows* point to neuronal cells. **h** and **i** TS olfactory bulb 8 and 33 days p.i. with HSV-1 Mckrae. **j** TS Brain Stem mock-infected control. **k** and **l** TS brain stem 8 and 33 days p.i. with HSV-1 McKrae. **m** TS cerebral cortex mock-infected control. **n** and **o** TS cerebral cortex 8 and 33 days p.i. with HSV-1 McKrae. **p** TS Thalamus mock-infected control. **q** and **r** TS thalamus 10 and 33 days p.i. with HSV-1 McKrae. **s** TS hippocampus mock-infected control. **t** and **u** TS hippocampus 10 and 33 days p.i. with HSV-1 McKrae, *arrows* show neurons positive for HSV-1 antigens. At mock-infected and 33 days p.i. with HSV-1 McKrae, TS olfactory bulbs, brain stem, cerebral cortex, thalamus and hippocampus showed no HSV-1-positive signal

To further characterize the tree shrew CNS infection, we performed immunohistochemistry to detect HSV-1 antigens during the course of infection. MRI 3D reconstructions of the tree shrew brains in dorsal, ventral, and left side view with dissected tissues circled in red are shown in Fig. 4 (Zheng et al. 2014). HSV-positive signals were detected from olfactory bulbs and brainstem of McKrae-infected mice at 5 days p.i. (arrows in Fig. 5b, e), while in Mock-infected samples, no positive signals were observed (Fig. 5a, d). The mouse brain stem and olfactory bulb are two brain regions where HSV-1 has previously been detected (Chen et al. 2006). Compared with mice, HSV-1 antigen signals in infected tree shrew brains appear from 8 days p.i. and become more significant at 10 days p.i. Strong and robust signals were detected in the olfactory bulbs and brainstem, but we also observed HSV-1 antigens in cerebral cortex, hippocampus, and thalamus at 8 and 10 days p.i. (see Fig. 5h, k, n, q, and t, arrows labels positive signals). No HSV-1 antigen signals were seen in any brains regions of the mock-infected animals (Fig. 5g, j, m, p, and s). There was no specific region where the virus appeared first, suggesting that once it entered the brain, the virus spread quickly through neuronal connections. However, based on the abundance of HSV-1 antigen signals and the route of infection, the brain stem and olfactory bulb may be the portals where the virus enters the CNS. After the acute stage of infection, the CNS of the infected mice and tree shrews showed no IHC signals using HSV-1 antibodies (Fig. 5c, f, i, l, o, r, and u). Our mouse CNS results are consistent with observations by others (Rock and Fraser 1983; Smith et al. 2000). These results indicated that there is no persistent infection after the acute stage in the CNS tissue of either mice or tree shrews.

### Immunofluorescence detected HSV-1 ICP4 proteins in tree shrew brain

**Fig. 6:**
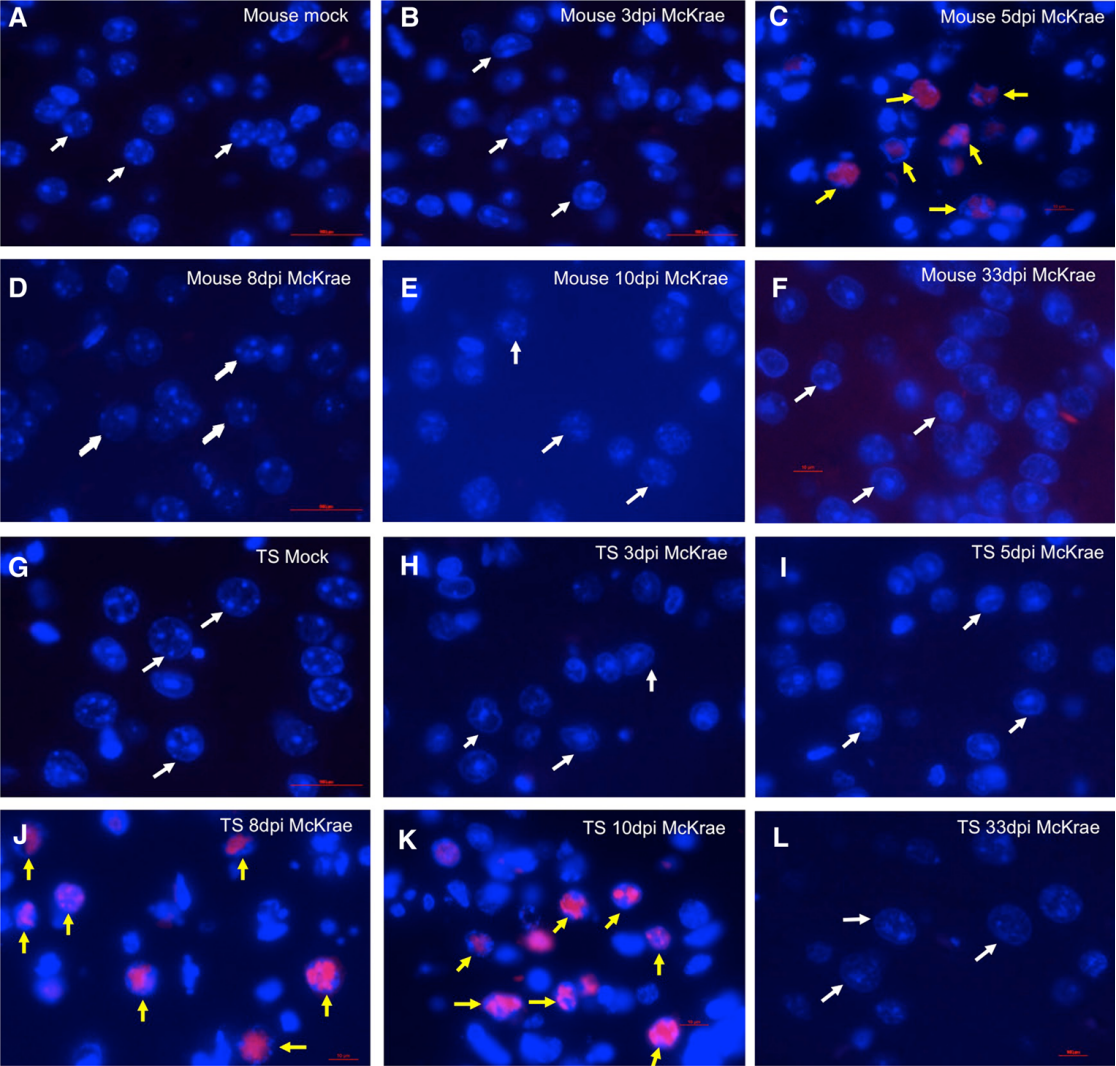
Immunofluorescent staining of ICP4 protein in mouse and tree shrew brain. At 3, 5, 8, 10, and 33 days p.i. with HSV-1 strain McKrae, mock and infected mice and tree shrews were euthanized, brains dissected, paraffin embedded and sectioned on a microtome before staining with an immunofluorescent ICP4 antibody. **a** Mock-infected mouse brain control, no positive signal detected, *white arrows* indicate neuronal cells. **b** HSV-1 McKrae infected mice at 3 days post-infection showed no ICP4 signal. **c** At 5 days p.i., ICP4 signal was readily detected in large neuronal cells in the brain. *Yellow arrows* point to ICP4-positive signal. **d** At 8 days p.i., ICP4 staining showed no signal. **e** At 10 days p.i., ICP4 stained showed no signal. **f** At 33 days p.i., ICP4 stained showed no signal. **g** Mock-infected tree shrew brain control, *white arrows* point to neuronal cells. **h** Infected tree shrews brain at 3 days p.i. shows no ICP4 signal. **i** Infected tree shrew brain at 5 days p.i. shows no ICP4 signal. **j** HSV-1 McKrea infected tree shrew brain section at 8 day p.i., showing positive ICP4 signal. **k** At 10 days p.i., more robust positive ICP4 signals were seen. **l** At 33 days p.i., ICP4 stained was no signal

The qRT-PCR and immunohistochemistry results (Figs. 3 and 5) suggest that HSV-1 underwent a productive infection and therefore should be expressing immediate early gene proteins. To confirm this, we performed immunofluorescence with ICP4 monoclonal antibodies to detect the ICP4 protein. Figure 6a–e shows mock-infected and McKrae-infected mouse brain during the acute phase. We could clearly see ICP4 staining appear at 5 days post-infection in the nucleus of neuronal cells (yellow arrows in Fig. 6c). In mock-infected mouse brain, or in 3, 8, and 10 days post-infection mouse brain, no ICP4 signal was seen (white arrows point to neuronal nucleus in Fig. 6a, b, d, and e). Figure 6f shows no ICP4 signal in mouse brain at 33 days p.i. In infected tree shrew brains, robust positive ICP4 protein signals were first seen at 8 days p.i. (yellow arrows in Fig. 6j) and remain robust at 10 days p.i. (Fig. 6k). In mock-infected tree shrew brain, or in 3, 33 days post-infection tree shrew brain, no ICP4 signal was seen (white arrows point to neuronal nucleus in Fig. 6g, h, and l). Unlike mouse, there was no ICP4 signal prior to 5 day p.i. in tree shrews (Fig. 6i). The delayed appearance of ICP4 compared to mouse is consistent with the qRT-PCR experiment and IHC staining described above (Figs. 3 and 5), suggesting that the virus may be taking a longer path or different route entering the CNS.

### In situ hybridization detected LAT transcription in HSV-1-infected tree shrew brain

**Fig. 7:**
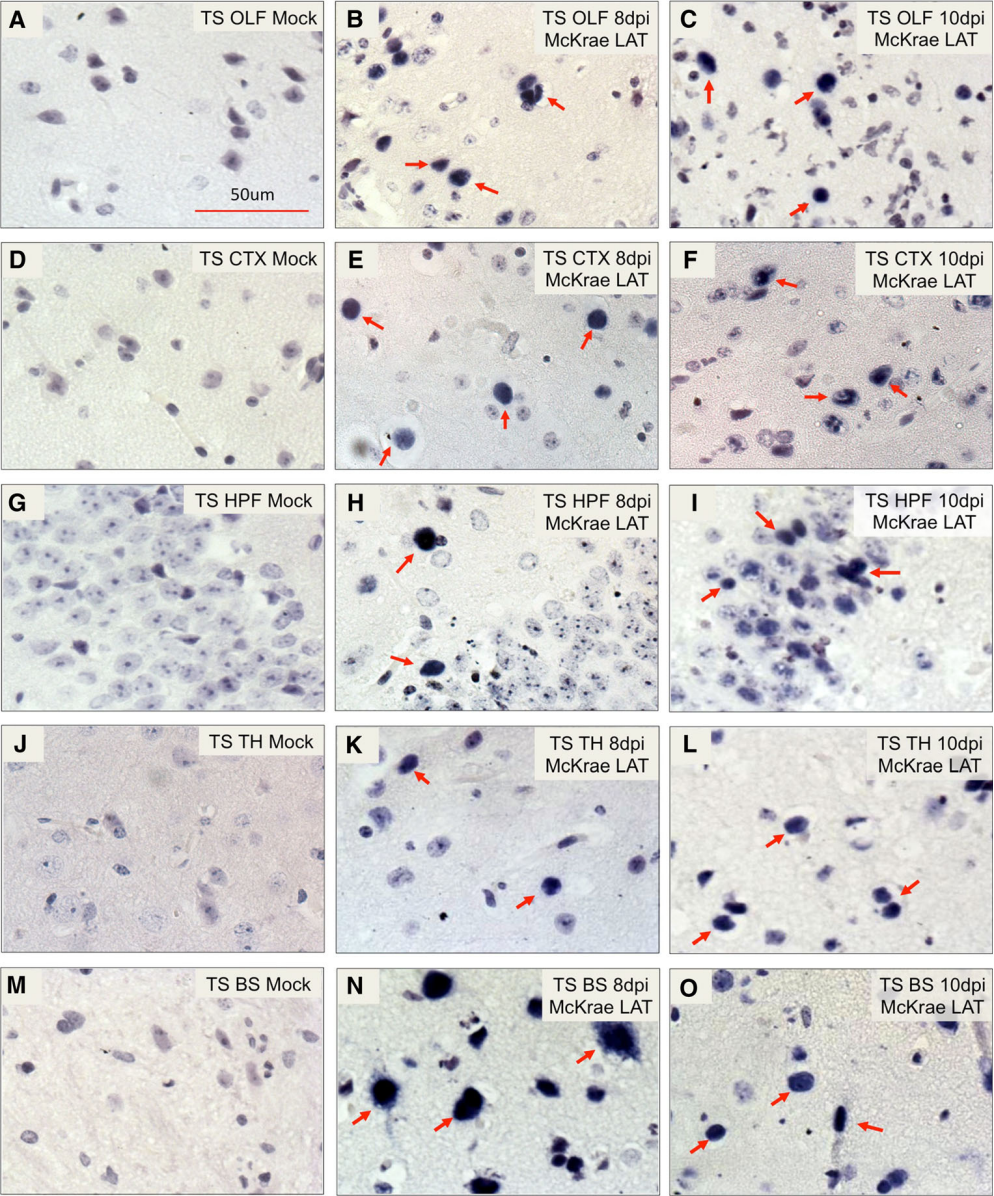
Detection of LAT signals in HSV-1 acute infected tree shrew brain by in situ hybridization. Sections of tree shrew brain, embedded in paraffin, were used to perform RNA in situ hybridization, as described in the “Methods” using RNA probes corresponding to the LAT intron region. Each panel shows representative staining from groups of between 3 and 6 animals. **a** Mock-infected tree shrew olfactory bulb (OLF) control showed no signal. **b** and **c** By days 8 and 10 p.i., LAT signal was readily detectable in tree shrew OLF (*red arrow*). **d** Mock-infected tree shrew cerebral cortex (CTX) control showed no signal. **e** and **f** By days 8 and 10 p.i., LAT signal was detectable in tree shrew CTX (*red arrow*). **g** Mock-infected tree shrew hippocampal formation (HPF) control showed no signal. **h** and **i** At days 8 and 10 p.i., LAT signal was detectable in tree shrew HPF (*red arrow*). **j** Mock infected tree shrew thalamus (TH) control showed no signal. **k** and **l** By days 8 and 10 p.i., LAT signal was detectable in tree shrew TH (*red arrow*). **m** Mock-infected tree shrew brain stem (BS) control showed no signal. **n** and **o** By days 8 and 10 p.i., LAT signal was detectable in tree shrew BS (*red arrow*)

As LAT is the only transcript consistently expressed during HSV latency from a large number of studies (Bloom 2004), its expression in the tree shrew brain is indicative that HSV-1 may be latently infecting the tree shrew CNS. To definitively confirm the existence and distribution of LAT transcript, we used RNA probes corresponding to the stable HSV-1 LAT intron region (probe position: 120,384–121,418 nt) and performed in situ hybridization as described in the infected tree shrew brain. As controls, mock-infected tree shrew CNS sections were subject to in situ hybridization, and no positive signal could be seen in any brain regions of these animals (Fig. 7a, d, g, j, and m). In the infected group, LAT signal could be detected in olfactory bulbs, cerebral cortex, hippocampus, thalamus, and brainstem of infected tree shrews as early as 8 days p.i. (see arrows in Fig. 7b, e, h, k, and n). At the same brain region of 10 days p.i., more LAT signals were detected (arrows in Fig. 7c, f, i, l, and o).

**Fig. 8:**
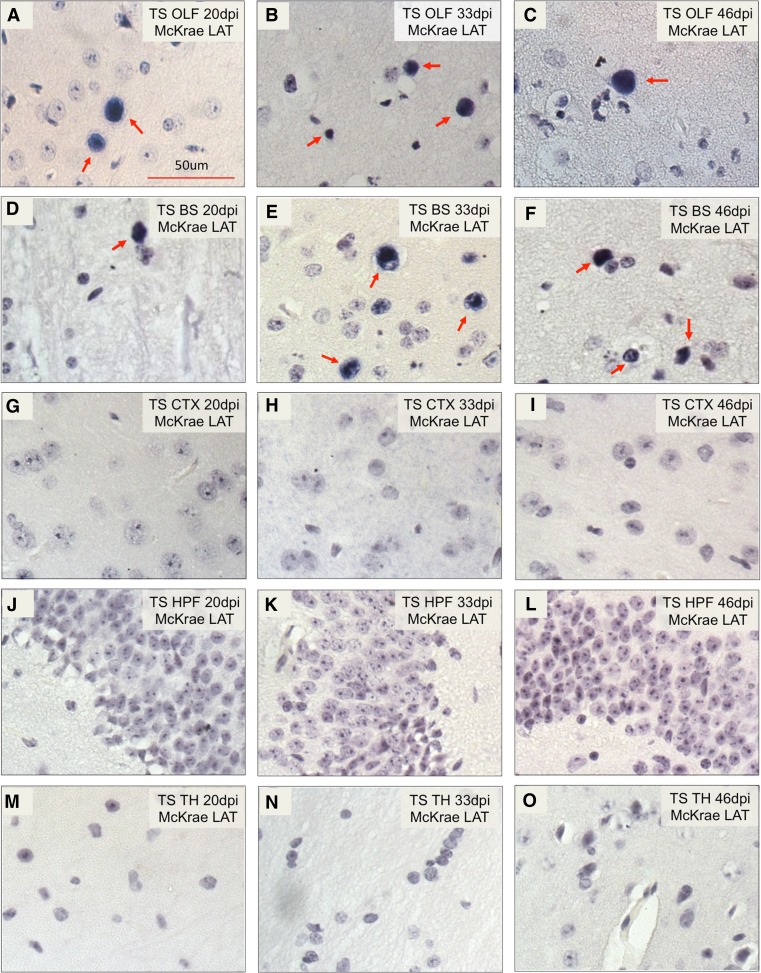
Detection of LAT signals in HSV-1 latent infected tree shrew brain by in situ hybridization. LAT intron signals were detected in olfactory bulb (OLF), brain stem (BS), cerebral cortex (CTX), hippocampal formation (HPF) and thalamus (TH) at 20, 33 and 46 days p.i. **a**–**c** By days 20, 33, and 46 p.i., LAT signal was detectable in tree shrew OLF (*red arrow*). **d**–**f** and in tree shrew BS (*red arrow*). **g**–**i** By days 20, 33, and 46 p.i., no LAT signal was detectable in tree shrew CTX. **j**–**l** Tree shrew HPF **m**–**o** or in tree shrew TH

We next determined LAT expression after the acute stage and detected LAT signals mostly in the tree shrew olfactory bulbs and brainstem at 20, 33, and 46 days p.i. (arrows in Fig. 8a–f). In the cerebral cortex, hippocampus and thalamus at 20, 33, and 46 days p.i., no detectable LAT transcription was seen (8G-8O). We noted that these LAT signals are nuclear, consistent with its distribution in human and other animal models of HSV latency, and its role as a noncoding RNA (Umbach et al. 2008). Since the probe was made against the intron region of LAT, the strength of the signal also supports high stability of the intron in tree shrews, as previously reported in mice (Farrell et al. 1991). Taken together, the in situ hybridization results discussed here, and qRT-PCR study in Fig. 3 strongly suggests that HSV-1 establishes a latent infection in the tree shrew brain.

### Tree Shrews brains are latently infected as determined by the explant cocultivation test

**Fig. 9:**
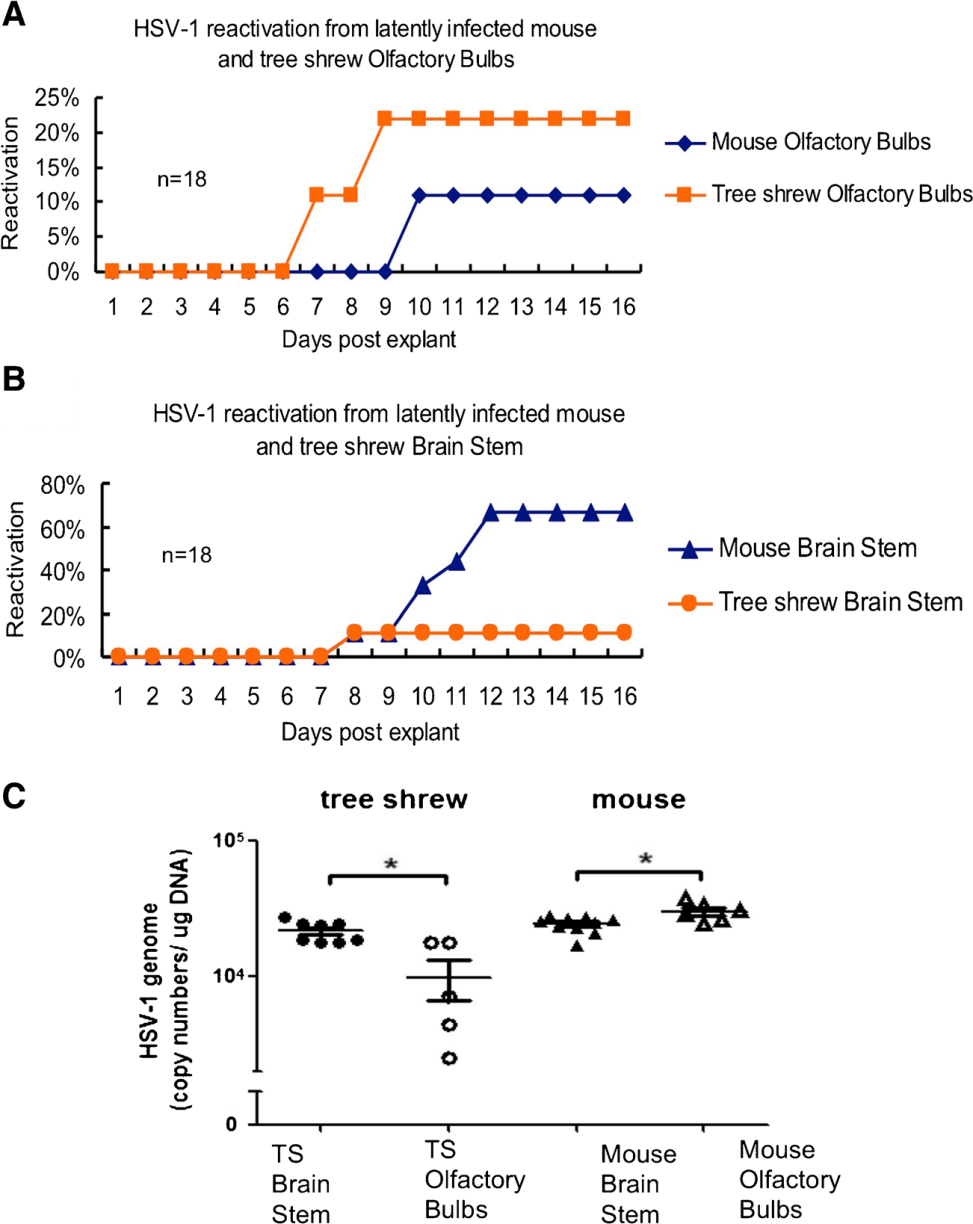
HSV-1 reactivation from infected mice and tree shrews brain. Mice and tree shrews were infected with HSV-1 strain McKrae at the indicated dose via eye cornea inoculation. At 30 days post-infection (dpi), neural tissues were harvested and assayed for reactivation of latent virus as described in the “Methods.” Data were collected from at least two independent experiments. The number of animals assayed in each group was 18. **a** Olfactory bulb, *blue rhombus* represented mouse (11 % reactivation). *Orange square* represented tree shrew olfactory bulb (22 % reactivation). **b** Brain stem, *blue triangle* represented mouse (67 % reactivation); *orange circles* represented tree shrew (11 % reactivation). **c** Viral DNA load in latent mouse and tree shrew olfactory bulbs and brain stems. Brain stems and olfactory bulbs of tree shrews or mice infected with HSV-1 McKrae for 30 days were dissected to quantify the numbers of copies of viral genomes per μg of tissue DNA. Each point on the scattergrams represents an individual sample, and the *horizontal lines* represent the mean values for each group. *P < 0.05; Mann–Whitney *U* test

The data described above suggests that HSV-1 latently infects the tree shrew CNS. To support this hypothesis, an explant co-cultivation test was performed as described by Chen et al. (2006). The explant cocultivation test using HSV-1 latently infected trigeminal ganglia has been widely used for many years to demonstrate viral reactivation from latency in PNS tissue (Stevens and Cook 1971). However, this test has failed on several occasions to show reactivation from CNS tissue of mice. Two possibilities could account this difference, either the HSV-1 genome was permanently silenced or the higher lipid content of CNS tissue block the reactivated virus from entering culture medium to infect the RS1 cells. Many years ago, Knotts et al. reported explants cocultivation of HSV from latent rabbit brain (Knotts et al. 1973), and more recently, Chen SH et al. described a modified method, which included a step to dissociate neurons and demonstrated that HSV-1 indeed could be reactivated from the infected brain, thus confirming that HSV-1 could latently infect the CNS (Chen et al. 2006). Using this modified explant cocultivation method, infectious HSV-1 virus was recovered from olfactory bulbs and brainstem of latently infected mice and tree shrews. In the tree shrew, we recovered reactivated virus from olfactory bulbs of 22 % of the tested animals, while in mouse the cumulative rate of recovery was 11 % (Fig. 9a). The appearance of CPE, a sign of reactivated virus infecting a monolayer-cultured cells, was 7 days for tree shrew olfactory bulbs and 10 days for the mouse counterpart. Both of these times are significantly delayed compared trigeminal ganglia explant cocultivations. In the brain stems, the rate of reactivation was reversed, with mouse showing a much higher rate of reactivation at 67 %, while tree shrew brainstem at 11 %. The first reactivation in tree shrew is also sooner than that of mouse, with tree shrew at 8 days, and mouse at 8–12 days (Fig. 9b). These results strongly support the hypothesis that HSV-1 indeed latently infects the tree shrew CNS.

To distinguish between persistent and latent infection, brain tissue from each animal was dissected, homogenized, centrifuged at slow speed, and the supernatant incubated with RS1 cells for over 10 days. No infectious virus was recovered from the brain supernatants of tested animals, although the viral DNA is clearly present in both the mouse and tree shrew brain tissues (Fig. 9c). These results are consistent with the IHC data (Fig. 5), immunofluorescent staining using ICP4 antibody (Fig. 6) and previous studies (Cabrera et al. 1980; Yao et al. 2012). They confirm that a persistent infection was not present in the neural tissues used for our reactivation studys, and thus, the HSV-1 virus was latent in both mice and tree shrews.

## Discussion

Here, we described infection of the tree shrew brain following cornea inoculation with the pathogenic HSV-1 McKrae strain. During the acute phase of infection, the most severely infected tree shrews exhibited many symptoms similar to viral encephalitis in humans, manifested by anorexia, lethargy, ataxia, torticollis, and abnormal whirl before perishing during the acute stage. Homogenates of brain tissue from infected tree shrew and mouse contain infectious virus indicating an acute infectious stage that started shortly after inoculation and lasted approximately 2 weeks. qRT-PCR analyses detected viral IE genes ICP0, ICP4 transcripts, and immunohistochemistry using anti HSV-1 antibodies and immunofluorescence with anti ICP4 antibody confirmed the existence of productive infection in both mouse and tree shrew brains. After the acute stage of infection, the LAT transcript continues to be expressed in the absence of ICP4, especially in tree shrew brain, suggesting that the virus is latent. Homogenizing the brain tissue and testing the supernatants for virus on tissue culture cells confirmed this. Using a modified explant cocultivation method (Chen et al. 2006), we found that virus could indeed be reactivated from these latently infected brain tissues. However, the frequency was lower and the appearance of CPE delayed compared to latently infected mouse and tree shrew TGs.

### Route of viral entry into the CNS

Following eye infection, HSV is transmitted via fast axional transport to neuron cell bodies in the trigeminal ganglion, where it can form latent infections and be transmitted retrograde to the nuclei of the trigeminal ganglion in the brain stem. It has been speculated that, following eye inoculation, HSV-1 can enter the nasal passage via the canaliculus and lacrimal sac from which it is transferred to the nasolacrimal duct and into the back of the nose. This is the route tears take when draining from the eye. This virus can then have direct access to CNS neurons in the back of the nose and enter the olfactory bulb (Tullo et al. 1982; Spivack and Fraser 1987; Deatly et al. 1988). Virus may also enter the CNS following reactivation of latent virus in the PNS and subsequent retrograde transport to the nuclei of the trigeminal nerve in the brain stem. In the infection models we used, we observed high levels of infectious virus and viral proteins from both olfactory bulb and the brain stem. This observation suggests that both routes of CNS entry exist in tree shrew and mouse. However, comparing the explant cocultivation results from brain stem and olfactory bulb between mouse and tree shrew, the tree shrew appeared to have a higher frequency of reactivation from the olfactory bulb, while mouse seemed to have a higher frequency from brain stem tissue. This difference suggests that the route of entry into the CNS in the tree shrew is predominately through the olfactory bulb, while in mouse HSV-1 more likely enters the CNS through the brain stem. The delay of viral appearance in the CNS in the tree shrew may also support this hypothesis, or may be due to the difference in distances the virus has to transverse in the two different sized animals.

### HSV-1 latency in the tree shrew brain

HSV-1 latency is well documented in infected ganglia (Spivack and Fraser 1987; Spivack et al. 1995; Webre et al. 2012; Roizman and Whitley 2013), but whether it also latently infects the CNS, as determined by reactivation from CNS tissue, is an important question that has seen data both supporting and not supporting reactivation. This question is key to our understanding of the pathogenesis of Herpes Simplex encephalitis. Explant cocultivation of latently infected ganglia with monolayers of susceptible cells has been widely adopted by the field as a test for reactivation from latency (Block et al. 1990). Such a method has not been widely successful to show HSV-1 reactivation from infected CNS tissue and is one of the main reasons for the notion that HSV-1 cannot be reactivated from CNS tissue and may be permanently silenced in the CNS (Cabrera et al. 1980). Recently, Chen et al. have demonstrated that HSV-1-infected mouse CNS tissue can produce infectious virus using a modified explant cocultivation method (Chen et al. 2006). Using this method, we were able to recover live infectious HSV-1 virus from infected tree shrew brain tissue. These findings support the hypothesis that the HSV-1 genome is not permanently silenced and can be reactivated under appropriate conditions. Based on the expression of LAT, the absence of detectable virus and viral proteins of infected brain tissue beyond the acute stage of infection, and the ability to reactivate from explant, we conclude that HSV-1 established latent infection in tree shrew CNS. Whether HSV-1 latency in the CNS is the same as the latency in trigeminal ganglia, and whether latent HSV-1 can be reactivated under physiological conditions remains to be demonstrated. Clearly, while humans commonly show recurrent reactivations at the periphery, they rarely show signs of encephalitis.
